# Influence of Introgression and Geological Processes on Phylogenetic Relationships of Western North American Mountain Suckers (*Pantosteus*, Catostomidae)

**DOI:** 10.1371/journal.pone.0090061

**Published:** 2014-03-11

**Authors:** Peter J. Unmack, Thomas E. Dowling, Nina J. Laitinen, Carol L. Secor, Richard L. Mayden, Dennis K. Shiozawa, Gerald R. Smith

**Affiliations:** 1 WIDB 401, Department of Biology, Brigham Young University, Provo, Utah, United States of America; 2 National Evolutionary Synthesis Center, Durham, North Carolina, United States of America; 3 School of Life Sciences, Arizona State University, Tempe, Arizona, United States of America; 4 Department of Biological Sciences, Wayne State University, Detroit, Michigan, United States of America; 5 Department of Biology, Saint Louis University, St. Louis, Missouri, United States of America; 6 Museum of Zoology, The University of Michigan, Ann Arbor, Michigan, United States of America; Team ‘Evo-Devo of Vertebrate Dentition’, France

## Abstract

Intense geological activity caused major topographic changes in Western North America over the past 15 million years. Major rivers here are composites of different ancient rivers, resulting in isolation and mixing episodes between river basins over time. This history influenced the diversification of most of the aquatic fauna. The genus *Pantosteus* is one of several clades centered in this tectonically active region. The eight recognized *Pantosteus* species are widespread and common across southwestern Canada, western USA and into northern Mexico. They are typically found in medium gradient, middle-elevation reaches of rivers over rocky substrates. This study (1) compares molecular data with morphological and paleontological data for proposed species of *Pantosteus*, (2) tests hypotheses of their monophyly, (3) uses these data for phylogenetic inferences of sister-group relationships, and (4) estimates timing of divergence events of identified lineages. Using 8055 base pairs from mitochondrial DNA protein coding genes, *Pantosteus* and *Catostomus* are reciprocally monophyletic, in contrast with morphological data. The only exception to a monophyletic *Pantosteus* is *P. columbianus* whose mtDNA is closely aligned with *C. tahoensis* because of introgression. Within *Pantosteus*, several species have deep genetic divergences among allopatric sister lineages, several of which are diagnosed and elevated to species, bringing the total diversity in the group to 11 species. Conflicting molecular and morphological data may be resolved when patterns of divergence are shown to be correlated with sympatry and evidence of introgression.

## Introduction

**Figure 1:**
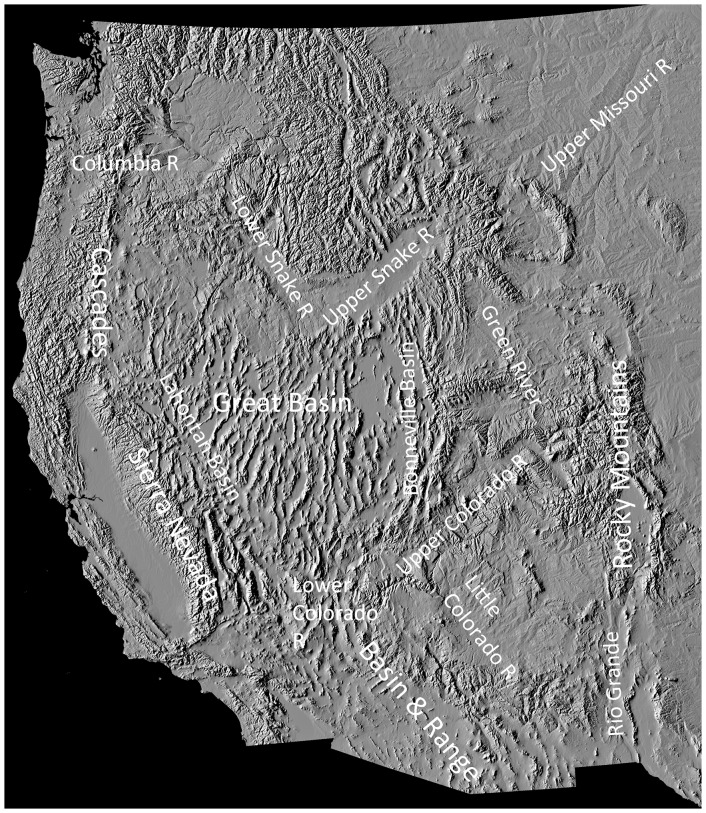
Major Late Cenozoic topographic features, drainage basins, and rivers responsible for isolation and evolution of *Pantosteus*. The hydrographic Great Basin, between the Sierra Nevada and the Colorado Plateau, is the northern, much-extended section of the Basin and Range province, which continues south into Mexico and east to the Rio Grande Rift.

Western North America experienced intense geological activity resulting in major topographic changes over the past 15 million years, during which time the modern freshwater fish fauna of the region evolved [1], [2]. The North American continental crust moved westward over subducting plates, and was extended (stretched) by nearly 100 percent, causing development of about 170 mountain ranges ca 100 km long, separating narrow, north-trending basins (Fig. 1). These roughly parallel basins extend from southern Oregon and Idaho, across Nevada and western Utah into Mexico. The Great Basin is flanked on the west by the Sierra Nevada and on the east by the Colorado Plateau and Rocky Mountains [3], [4]. Changes in elevation, caused by major north-south normal faults, resulted in hundreds of short, swift mountain streams often ending in isolated basins. These geological changes caused the diversification of lineages of many aquatic organisms [5], [6].

The evolution of basin and range topography caused isolation, which permitted diversification within the aquatic fauna [7], [8]. Tectonism and volcanism modified drainage patterns to form several huge, long-lived lakes such as Miocene Chalk Hills Lake [9] and Pliocene Glenns Ferry Lake in southwest Idaho [10], late Miocene Hopi Lake in northwest Arizona [11], Pleistocene Lake Bonneville in Utah [12]–[14], Pleistocene Lake Lahontan and associated lakes in Nevada [15] and southern Oregon [16], Pleistocene Tulare Lake, California [17], and many other fish-inhabited lakes in the Great Basin [7], [18]. These sometimes-isolated basins were the original crucibles of local evolution of subspecies and species of various fishes in the Miocene and Pliocene [6].

Today, the major rivers of western North America are composites of more ancient rivers. The Colorado River flows southwestward across the Colorado Plateau through Grand Canyon to the Grand Wash fault zone, where the river enters the Great Basin and adds to its fish fauna [19], [20]. North of the Great Basin, the Snake River flows across southern Idaho to Hells Canyon, where it was captured in the late Pliocene by the Columbia River [21], [22]. The Bear River flows north from the Uinta Mountains to southeast Idaho, where it was diverted south into the Bonneville Basin about 100 Ka [23], causing the Bonneville Basin, Utah, to overflow and become tributary to the Snake and Columbia rivers in Idaho. In the Miocene the Lahontan Basin, Nevada, flowed west to the Pacific Ocean and later overflowed to the Upper Snake River [6], [15]. Uplift of the Sierra Nevada and rise of the Cascade Mountains changed climates across the Great Basin [24], [25] and influenced drainage connections. Each of these geological and hydrologic events is hypothesized to have influenced evolution of fish populations to be discussed (Fig. 1).

The geological and climatic history of the west also caused high extinction rates, resulting in a depauperate fish fauna relative to other areas of North America [26]. Currently restricted basins have fewer than 25 native fishes and high endemism, typically between 25 and 74% [27]. The Rocky Mountains and associated mountain ranges of the continental divide [2] have largely isolated western rivers from species-rich drainages in the tectonically stable eastern USA. Some western fish clades have Paleogene origins and represent endemic lineages whose evolution is consistent with the active geologic history of this region. Examples include western clades in the Salmonidae, Catostomini [6], Cyprinidae [28], [29], Cyprinodontidae [30], Goodeidae [31], [32] and *Cottus* [33]. Additionally, evidence has been provided for frequent episodes of introgression of some fishes [34], [35], resulting in complete mtDNA replacement [36], [37], and the evolution of species of hybrid origin [37], [38].

**Figure 2:**
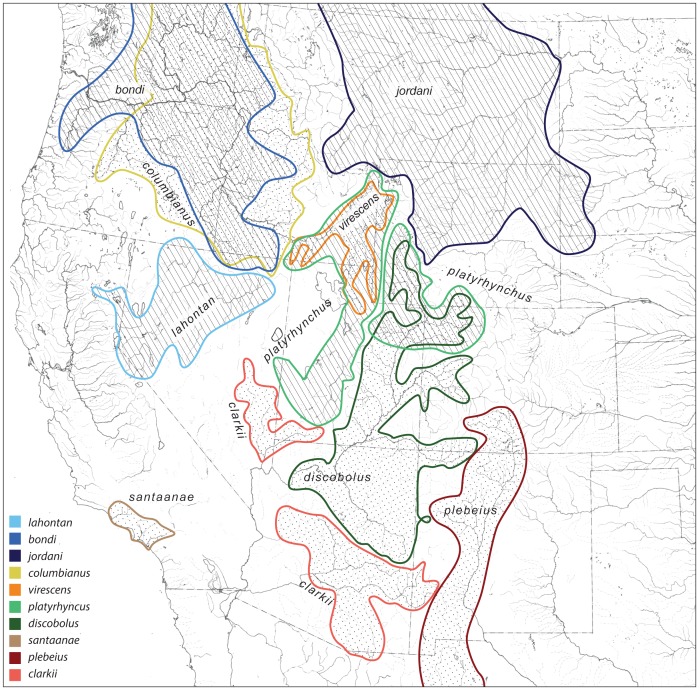
Distribution of the 11 species of *Pantosteus*. Border species are found north and south of the margins of this map.

Mountain suckers (*Pantosteus*, sometimes considered a subgenus of *Catostomus*) is one of several clades centered in the tectonically active part of western North America. Species of this lineage occur from southwestern Canada, western USA, and into northern Mexico, inhabiting middle-elevation reaches of rivers of the Basin and Range, Coast Ranges, Cascade Mountains, Rocky Mountains, east to the Black Hills and Sierra Madres [20], [34] (Figs. 1, 2). These cool-water, benthic fishes were investigated by early western American ichthyologists, from Cope in 1872 [39] to Snyder in 1924 [40]. Smith [34] presented a classification recognizing six polytypic species of *Pantosteus* (distributions in Fig. 2). The four southern forms included *P. plebeius* in the Rio Grande Basin and its major tributary the Rio Conchos, the Rio Nazas and several Pacific basins in Mexico, *P. santaanae* in the Los Angeles Basin, *P. clarkii* in the Lower Colorado Basin below Grand Canyon, including the Pluvial White, Virgin, Bill Williams and Gila basins, and *P. discobolus* in the Upper Colorado Basin, Upper Snake River and northern Bonneville Basin. The northern group consists of *P. platyrhynchus* in the Great Basin, Columbia-Snake, Fraser, upper Saskatchewan, Missouri and Green drainages; and *P. columbianus* in the Columbia-Snake drainage. All species except *P. santaanae* exhibit geographic variation, with subgroups that include morphologically differentiated populations in adjacent drainages. Some species contain variants recognized as subspecies: *P. discobolus jarrovii* in the Zuni River, New Mexico, and other drainages in northeast Arizona [41], *P. columbianus hubbsi*, in the Wood River drainage, Idaho [34], and *P. columbianus palouseanus* in the Palouse River, Washington [34]. An additional species, *P. nebuliferus*, from the Nazas Basin, was recognized by [42].

Smith and Koehn [43] conducted a phylogenetic analysis of 16 species of *Catostomus* and *Pantosteus* using both morphological and biochemical data, yielding two general groups in *Catostomus*: (1) a lineage with large, low-elevation, slow-water species in a paraphyletic subgenus *Catostomus*, and (2) a lineage of smaller mountain suckers adapted to cooler and swifter rivers at higher elevations. *Pantosteus columbianus* has traits diagnostic for both *Catostomus* and *Pantosteus*. Smith and Koehn [43] and [20] hypothesized that this taxon is a product of introgressive hybridization. One study has used DNA sequences to examine broader relationships among catostomine species. Doosey et al. [44] examined six species of *Pantosteus* and recovered five of them in a monophyletic group nested within *Catostomus*. The only species of *Pantosteus* excluded from this lineage was *P. columbianus*.

The above studies illustrated how complex distribution of traits can impact the ability to delimit taxa and interpret their evolutionary origins. This is especially true for *Pantosteus* and *Catostomus* as indicated by the proposed paraphyly of some taxa. The goals of the present work are to (1) compare molecular data with morphological and paleontological data for the proposed species of *Pantosteus*, (2) test hypotheses of their monophyly, (3) use these data for phylogenetic inferences of sister-group relationships, and (4) estimate timing of divergence events of identified lineages. With these insights into the reconstructed evolutionary history of the group, we evaluate the variation and distribution of traits found in *Pantosteus* lineages to revise the taxonomy of the genus and consider the impact of geological processes on diversity in this group.

## Materials and Methods

### Ethics Statement

Permission to undertake field work and collect specimens was obtained under the following permits: Colorado fisheries research permit 01-AQ908, Idaho fisheries research permits F-84-90, F 84-90-10, Nevada fisheries research permit S21662, Utah fisheries research permit 4coll78, Arizona fisheries research permit SP592500, Mexico scientific collection permit issued to Héctor Espinosa, Permiso de Pesca de Fomento 0765. Specimens were obtained under Brigham Young University Institutional Animal Care and Use Committee (IACUC) approval 070403 and 10-1202, Arizona State University IACUC approval 09-1018R, and Saint Louis University IACUC approval 2134.

### Study Taxa and Sampling

We conducted range-wide molecular surveys of each species and selected individuals that represented both geographic and genetic diversity within the genus (Table 1). Outgroup taxa to represent the broader diversity within Catostomidae [44], [45] include: *C. ardens*, *C. catostomus*, *C. commersonii* (GenBank accession AB127394.1), *C. insignis*, *C. macrocheilus*, *C. tahoensis*, *Cycleptus elongatus* (AB126082.1), *Hypentelium nigricans* (AB242169.1), *Minytrema melanops* (DQ536432.1), *Moxostoma erythrurum* and *M. poecilurum* (AB242167.1) (Table 1).

**Table 1:** Locality data for all *Pantosteus* samples and outgroups.

species	locality	sub-basin	major basin
*Pantosteus clarkii*	White R	Pluvial White	L Colorado
*P. clarkii*	Virgin R	Virgin	L Colorado
*P. clarkii*	Francis Ck	Bill Williams	L Colorado
*P. clarkii*	Aravaipa Ck	Gila	L Colorado
*P. columbianus*	Salmon Falls Ck	L Snake	Columbia
*P. discobolus*	Rifle Ck	U Colorado (CO)	U Colorado
*P. discobolus*	Havasau Ck at mouth	L Colorado (AZ)	L Colorado
*P. discobolus*	Tsaile Ck	San Juan	U Colorado
*P. discobolus*	Wheatsfield Ck	San Juan	U Colorado
*P. discobolus*	East Clear Ck	Little Colorado	L Colorado
*P. discobolus*	Upper Little Colorado R	Little Colorado	L Colorado
*P. discobolus jarrovii*	Rio Nutria	Little Colorado	L Colorado
*P. jordani*	Whitewood Ck	Missouri (SD)	Mississippi
*P. jordani*	Lil Popo Agie R	Missouri (WY)	Mississippi
*P. lahontan*	McDermitt Ck	Quinn	Lahontan
*P. lahontan*	Lower Truckee R	Truckee	Lahontan
*P. lahontan*	E Walker R	Walker	Lahontan
*P. nebuliferus*	Arroyo Penon	Nazas	Nazas
*P. platyrhynchus*	Blackrock Ck	U Snake	U Snake
*P. platyrhynchus*	Bear R	Bear	Bonneville
*P. platyrhynchus*	Weber R	Weber	Bonneville
*P. platyrhynchus*	Soldier Ck	Spanish Fork	Bonneville
*P. platyrhynchus*	San Pitch R	Sevier	Bonneville
*P. platyrhynchus*	Salina Ck	Sevier	Bonneville
*P. platyrhynchus*	Mammoth Ck	Sevier	Bonneville
*P. platyrhynchus*	Fremont L	U Green	U Colorado
*P. plebeius*	S Fork Palomas Ck	Rio Grande	Rio Grande
*P. plebeius*	R Santa Clara	Santa Clara	Guzmán
*P. plebeius*	R Escalariado	Escalariado	Guzmán
*P. plebeius*	Arroyo Ureyna	Conchos	Conchos
*P. plebeius*	Arroyo Riito	Fuerte	Fuerte
*P. plebeius*	R Miravalles	Miravalles	Miravalles
*P. santaanae*	Big Tujunga Ck	Tujunga	Los Angeles Basin
*P. santaanae*	San Gabriel R	San Gabriel	Los Angeles Basin
*P. santaanae*	Santa Ana R	Santa Ana	Los Angeles Basin
*P. bondi*	Similkameen R	Columbia	Columbia
*P. bondi*	Willamette R	Willamette	Columbia
*P. bondi*	Salmon Falls Ck	L Snake	Columbia
*P. virescens*	Twin Ck	U Snake	U Snake
*P. virescens*	Tin Cup Ck	U Snake	U Snake
*P. virescens*	Goose Ck	U Snake	U Snake
*P. virescens*	Weber R	Weber	Bonneville
*P. virescens*	Echo Ck	Weber	Bonneville
*Catostomus ardens*	Strawberry Res (introduced)	Duchesne	U Colorado
*Catostomus catostomus*	Boundary waters	Lake Superior	Great Lakes
*Catostomus commersonii*	GenBank AB127394.1		
*Catostomus insignis*	Eagle Ck	Gila	L Colorado
*Catostomus macrocheilus*	Grimes Ck	L Snake	Columbia
*Catostomus macrocheilus*	Palouse R	L Snake	Columbia
*Catostomus tahoensis*	Walker R	Walker	Lahontan
*Cycleptus elongatus*	GenBank AB126082.1		
*Hypentelium nigricans*	GenBank AB242169.1		
*Minytrema melanops*	GenBank DQ536432.1		
*Moxostoma erythrurum*	Middle Fork Vermilion R	Mississippi (IL)	Mississippi
*Moxostoma poecilurum*	GenBank AB242167.1		

### DNA Isolation, Amplification, and Sequencing

Genomic DNA was extracted from muscle tissue using the DNeasy Tissue Kit (QIAGEN Inc., Chatsworth CA) or by phenol-chloroform extraction as described in [46]. Nine of the 13 mtDNA protein coding genes (ND1, ND2, ND4L, ND4, ND5, ND6, ATPase6/8, cyt*b* and partial sequence from COIII) were amplified, representing approximately half of the mitochondrial genome (8055 bp). We obtained a large proportion of the mitochondrial genome in order to be able to obtain robust phylogenetic relationships as using a small number of genes provided poor resolution [47]. We did not pursue nuclear gene sequencing due to the low resolution of nuclear genes in the same species of catostomids [48], which indicates that a large number of nuclear loci would be required to provide informative phylogenetic results. Single and nested PCR amplification strategies were used to obtain product for different gene combinations. Details of the primers and nesting combinations are in Figure S1. For nested PCR the first reaction size was 10 µL. This first PCR reaction was then diluted to 1∶49, and 1 µL of this product was added to a second 25 µL reaction. All other single reactions were 25 µL. Final concentrations for PCR components were as follows: 25 ng template DNA, 0.25 µM of each primer, 0.625 units of Taq DNA polymerase, 0.1 mM of each dNTP, 2.5 µL of 10X reaction buffer and 2.5 mM MgCl_2_. Amplification parameters were as follows: 94°C for 2 min followed by 35 cycles of 94°C for 30 s, 48°C for 30 s, and 72°C for 60 s (in the first nested reactions this was increased by 1 min per each thousand bp), and 72°C for 7 min. PCR products were examined on a 1% agarose gel using SYBR safe DNA gel stain (Invitrogen, Eugene, OR, USA) and purified using a Montage PCR 96 plate (Millipore, Billerica, MA, USA). Sequences were obtained via cycle sequencing with Big Dye 3.0 dye terminator ready reaction kits using 1/16th reaction size (Applied Biosystems, Foster City, CA, USA). Sequencing reactions were run with an annealing temperature of 52°C (following the ABI manufacturer's protocol), cleaned using Sephadex columns in MultiScreen 96 well assay plates (Millipore, Billerica, MA, USA), and then dried. Most sequences were obtained using an Applied Biosystems 3730 XL automated sequencer at the Brigham Young University DNA Sequencing Center. All sequences obtained in this study were deposited in GenBank, accession numbers KJ441082-KJ441387 and the sequence alignment was deposited in Dryad, doi:10.5061/dryad.51mm0.

### Analysis of Sequence Data

Sequences were edited using Chromas Lite 2.0 (Technelysium, Tewantin, Queensland, Australia) and imported into BioEdit 7.0.5.2 [49]. Sequences coding for amino acids were aligned by eye and checked via amino acid coding in MEGA 4.0.2 [50] to test for unexpected frame shift errors or stop codons. Editing resulted in 8055 base pairs representing the complete sequence for the nine genes, plus 22 bp of COIII. Phylogenetic analyses were performed under maximum likelihood (ML) using RAxML 7.2.8 [51], [52] by bootstrapping with 1000 replicates with the final best ML tree being calculated using the GTRGAMMA model on the CIPRES cluster at the San Diego Supercomputer Center. Maximum parsimony (MP) analysis was conducted with PAUP* 4.0b10 [53] using a heuristic search with 1000 random additions and TBR branch swapping. Tree lengths reported for MP include both informative and uninformative characters. Robustness of nodes for MP was estimated by bootstrapping with 1000 replicates using a heuristic search with 10 random additions and TBR branch swapping. The ML tree from the DNA analysis presented in this study was deposited in TreeBASE, accession number TB2:S15337, (http://purl.org/phylo/treebase/phylows/study/TB2:S15337). Average between-species genetic distances were calculated based on the proportion of shared differences (p-distance) using MEGA for each lineage within *Pantosteus*, *Catostomus* as a whole, and then the remaining outgroups as a whole.

### Morphology and Age Estimates

Eighty-seven morphological characters and eight fossil occurrences were taken from [20]. Calibration of the rate of evolution requires fossils to be assigned to specific branches based on synapomorphies shared by the fossils and recent taxa. This is achieved by cladistic interpretation of apomorphic characters for each fossil. These taxa are then represented as terminal taxa on specific branches. Confidence limits of estimates of times of cladistic branching have two primary sources of error: (1) fossils are unlikely to represent a lineage soon after its divergence from its sister lineage [54], and (2) both sister lineages do not necessarily possess new apomorphies of the lineages immediately after their initial genetic isolation. Fossils of both lineages should ideally contribute to estimation of age of a node, but both are rarely available in the record. For these reasons it is not obvious which end of the stem branch of a crown group should be assigned the age represented by the fossil. We choose to correct the age of the oldest fossil bearing synapomorphies of a lineage in question with the method of [54], which uses the density of the record of other fossils from that lineage to estimate the age of origin of the stem branch. The number of million-year time horizons or independent localities in which fossils of the lineage occur are applied in an equation that uses these data to estimate the probability that the oldest fossil represents the first (unobserved) appearance of the lineage [54]:
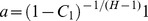
where *a* is the confidence interval as a fraction of the total known stratigraphic range, *C*_1_ is the 50% or 95% confidence level, and *H* is the number of known fossil horizons. The 50% interval is chosen as the mid-point of the distribution for calculation of the node calibration points. These were entered into the BEAST analyses. The ages of the oldest fossils, their identities, apomorphies, calibration points, locations, number of horizons, stratigraphy, and catalog numbers are given below, following best practice recommendations by [55].

**Figure 3:**
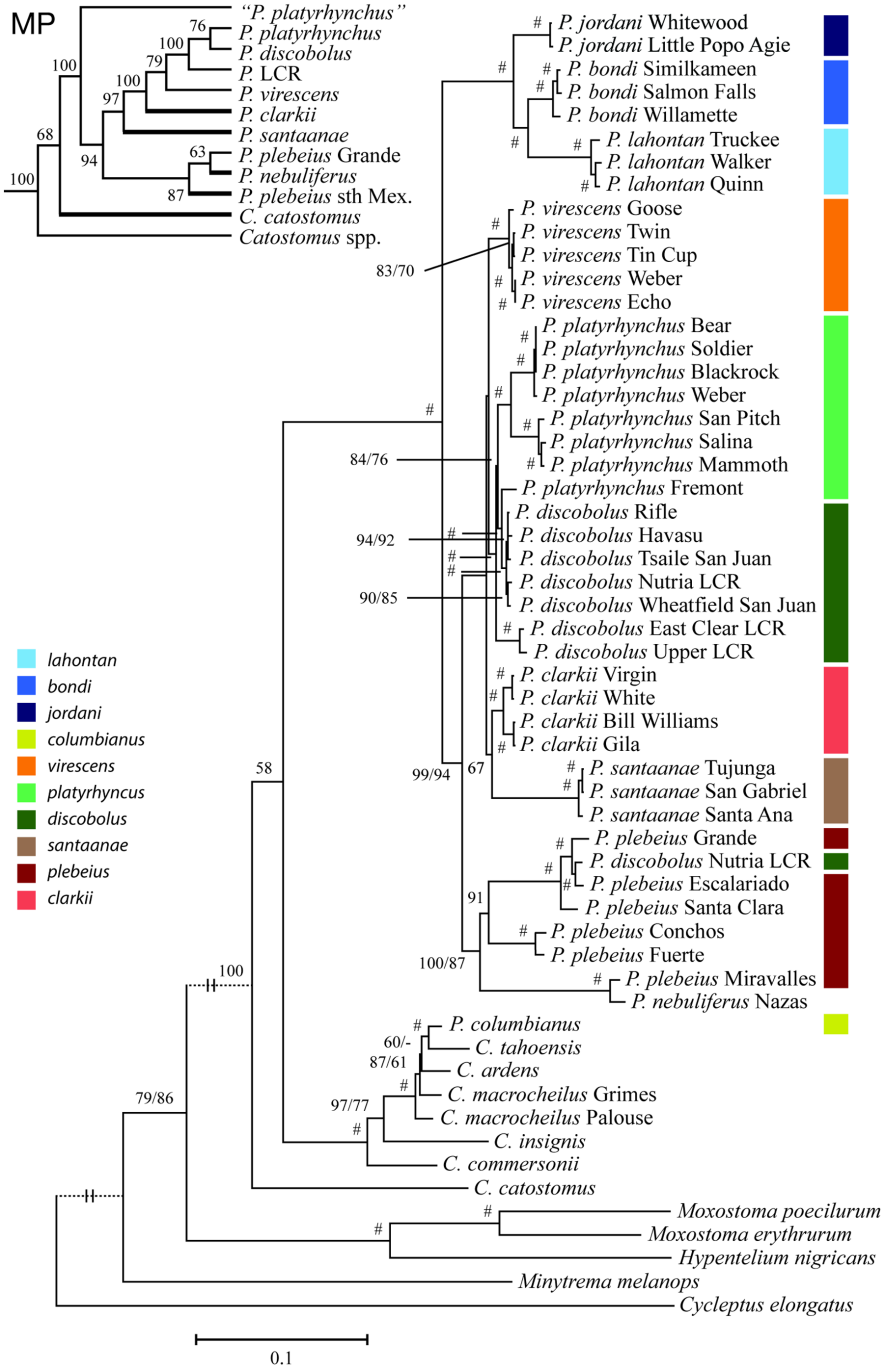
Maximum likelihood (ML) tree for *Pantosteus* based on analysis of mitochondrial DNA. A collapsed maximum parsimony (MP) topology is shown in the upper left corner, the thick branches indicate differences in topology to ML. All bootstrap values are based on 1000 pseudoreplicates, a # symbol represents bootstrap values over 95 for both ML/MP. Single bootstrap values are shown for nodes which are different to MP. The tree is rooted with *Cycleptus elongatus*. Locality details are provided in Table 1.

An important assumption is that the fish of interest could have lived in a depositional environment below the oldest fossil, so that the absence of fossils possessing synapomorphies of a specific lineage can be interpreted as absence of the lineage at that time. The probability that the earliest fossil occurrence represents the first occurrence following cladogenesis is then used to correct the age estimate. The corrected age is applied to the node joining the taxa bearing the morphological synapomorphies supporting their sister-group relationship (Fig. 3).

BEAST 1.7.1 [56] was used to estimate molecular divergence times of mtDNA lineages based on corrected fossil age estimates. We generated input files using BEAUti 1.7.1. The dataset was trimmed to single representatives per species/lineage because having a mix of within- and between-species data complicates dating owing to different processes for estimating within- versus between-species rates [57] (S. Ho, pers. comm.). The analysis used an uncorrelated lognormal relaxed molecular clock with rate variation following a tree prior using the speciation birth-death process, a GTR+I+G model (identified using the AIC in Modeltest 3.7, [58]). The topology was constrained to match the ML results.

Calibrations were based on eight time horizons [20], with age of occurrence estimated for two nodes with two date estimates in each based on [54]. These two dates, 5.5 Ma and 12.7 Ma were given a log-normal prior with a standard deviation of 1. BEAST analyses were run for 50 million generations, with parameters logged every 10000 generations. Multiple runs were conducted to check for stationarity and to ensure that independent runs were converging on a similar result. The log and tree files from four runs were combined in LogCombiner 1.7.1 with a 10% burn-in. The combined logfile was examined in Tracer 1.5, while the combined treefile was summarized using TreeAnnotator 1.7.1 with the mean values placed on the maximum clade credibility tree.

The diagnoses of recent and fossil forms with specimen disposition for taxa in the morphological phylogenetic analysis are detailed in [20]. Identities, localities, and curatorial data for 347 western catostomin skeletons can be accessed at the University of Michigan Museum of Zoology Fish Division website. The morphological tree was estimated with PAUP, using 87 partly new osteological and morphological traits defined and listed in matrix format in [20]. Inferred instances of introgression are plotted on the tree, based on observed scatter of non-congruent morphological and molecular character states. Alternative hypotheses (such as convergence and retained polymorphisms) were examined by contrasting molecular and morphological trees. High bootstrap support values were used to identify strongly supported nodes in each analysis. Conflict between nodes on these two trees allowed rejection of alternative hypotheses.

## Results and Discussion

### Phylogenetic Analyses using mtDNA Sequences

Sequence analysis of 58 specimens (Table 1) yielded 4777 invariant characters, 507 variable but parsimony uninformative characters, and 2771 parsimony informative characters. Maximum-likelihood analysis recovered one tree with a likelihood score of −60385.098771 (Fig. 3). Phylogenetic analysis of mtDNA sequences provided resolution among recognized species [20], [34] as well as identification of divergent lineages within four of the species. The *Pantosteus* mtDNA lineage was monophyletic, except that *P. columbianus* mtDNA was closely aligned with *C. tahoensis*, although it is morphologically in the *Pantosteus* clade. Both maximum likelihood and maximum parsimony methods recovered the same major lineages within *Pantosteus*, but with some different branching sequences among regional populations. In general, bootstrap analyses provide strong support (>95%) for most deep nodes (Fig. 3). In the following summary we only provide bootstrap values when support was less than strong (<95%). Mean p-distance between these 13 major lineages within *Pantosteus* varied between 2.2% and 10.3% (Table S1), while divergence between sister lineages varied between 2.2% and 8.3%. Outgroup lineages differed from *Pantosteus* by 11.4–19.2% pairwise sequence divergence (Table S1) except for the 2.7% p-distance between *C. tahoensis* and *P. columbianus*.

The earliest separation within the *Pantosteus* mtDNA clade is between the *P. discobolus*-*plebeius* clade and a clade (formerly part of *P. platyrhynchus*) comprising three groups of populations in the Columbia drainage, Missouri drainage and Lahontan Basin whose relationship is (Missouri (Lahontan, Columbia)). Sister to that lineage is the clade including all other *Pantosteus* species. The morphological *Pantosteus platyrhynchus* group has two forms of mtDNA on the tree–Bonneville Basin and Upper Snake River samples contain *P. discobolus* DNA, raising the possibility that the morphological traits are homoplasious (see below).

The next oldest mtDNA lineage in *Pantosteus* includes samples of southern forms (primarily from Mexico) that extend from the Nazas Basin north into the Rio Grande Basin and its past connectives. In the ML tree, the lineage including *P. nebuliferus* and *P. plebeius* from the Miravalles drainage was sister to a well-supported group of *P. plebeius* (93% bootstrap support) from southern (rios Conchos and Fuerte) and northern (Rio Grande and Guzman basins) lineages. In addition, a *P. plebeius* haplotype from the Rio Grande was found among samples taken from *P. discobolus jarrovii* populations from the upper Little Colorado River (Nutria Creek). This pattern is consistent with their history of introgression [41].

The remaining *Pantosteus* mtDNA lineages form a clade herein identified as the *P. discobolus* group (*sensu stricto*). This group consists of: *P. clarkii* from the Lower Colorado Basin, *P. santaanae* in the Los Angeles area, *P. discobolus* from the Upper Colorado Basin, *P. virescens* from the Weber, Bear, and Upper Snake rivers, and *P. platyrhynchus* from the Bonneville Basin, Upper Snake River, and upper Green River (Fig. 2). The *P. clarkii*-*P. santaanae* clade is sister to a clade including *P. discobolus*, *P. virescens*, and *P. platyrhynchus*. In this latter group, samples of *P. virescens* from the Weber, Bear, and Upper Snake rivers were sister to *P. discobolus* (Upper Colorado Basin) and *P. platyrhynchus* (Bonneville Basin). The high estimates of sequence divergence within the *P. discobolus* group suggest re-evaluation of the morphology of the Bonneville-Upper Snake populations, and leads to the recognition of these as *P. virescens* in this paper.

Morphological traits (gill-raker counts, vertebral counts, predorsal scale counts, lip papillae, sparse caudal interradial pigment, and deep caudal peduncles) of many individuals in the upper Green River suggest that these populations are similar to *P. platyrhynchus* from the Bonneville and Upper Snake drainages; however, individuals also possess scattered morphological traits of *P. discobolus* consistent with introgression between these species (see Fig. 22 in [20]). The presence of *P. discobolus* mtDNA in *P. platyrhynchus* in the Bonneville Basin renders the mtDNA lineages of these forms paraphyletic (Fig. 3). Samples of *P. platyrhynchus* mtDNA from the Bonneville Basin were subdivided into well-differentiated Sevier River and northern Bonneville-Upper Snake River lineages.

Maximum parsimony analysis of the mtDNA sequence data, with all characters weighted equally, recovered a single most parsimonious tree with a length of 11,151 (CI = 0.398, RI = 0.727). Two major topological differences exist between ML and MP trees; these are highlighted on a reduced MP tree (inset in Fig. 3). Among *P. plebeius* and relatives, the MP tree differs from the ML tree with *P. nebuliferus* and *P. plebeius* from the Rio Miravalles sister to the northern lineage of *P. plebeius* (found in only 63% of MP bootstrap replicates), with a southern group of *P. plebeius* sister to *P. nebuliferus* and Rio Grande-Guzman Basin lineages. The second difference in the MP tree involves species from the Bonneville, Colorado and Los Angeles basins, where *P. clarkii* rather than *P. santaanae* is the sister to the clade that includes all *P. discobolus* plus *P. platyrhynchus* from the Bonneville Basin. This placement of *P. clarkii* is found in 100% of the MP bootstrap replicates.

### Contrast of Molecular and Morphological Traits in *Pantosteus*

**Figure 4:**
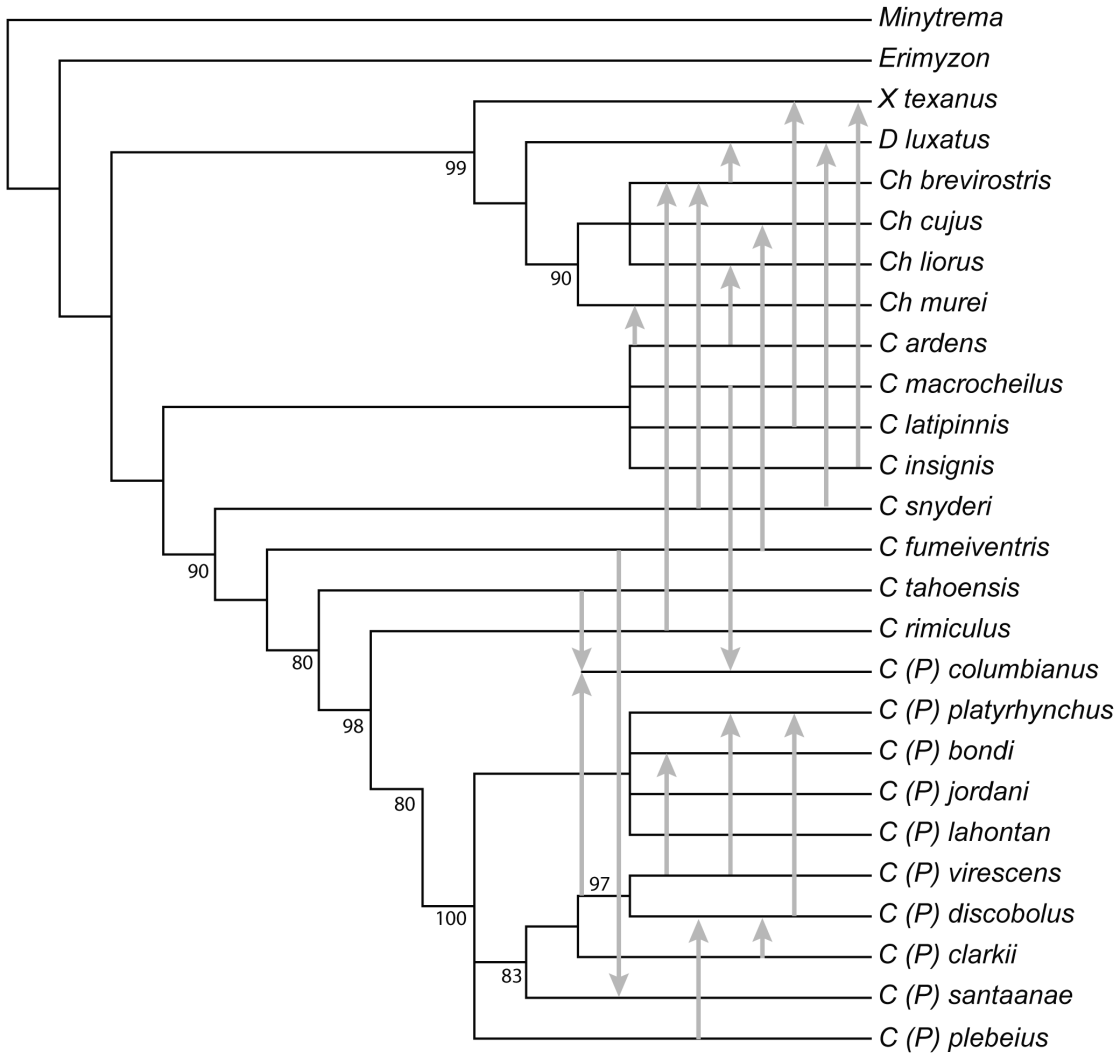
Morphology-based phylogenetic tree based on PAUP analysis of 87 characters of *Pantosteus* and other catostomin species from the genera *Xyrauchen*, *Deltistes*, *Chasmistes*, and *Castostomus*. Arrows indicate introgression of mtDNA and morphological traits (modified from [20]). Topology is based on the strict consensus of 63 equally shortest maximum parsimony trees (length = 252, CI = 0.53, RI = 0.83). Bootstrap values of >80 and above shown at nodes (from 1000 replicates).

*Pantosteus* Cope is a monophyletic group based on osteological and morphological characters (if evidence for introgression in *P. columbianus* and other extreme hybrids are excluded). *Pantosteus* is morphologically diagnosed by: dentaries with their anterior, distal process turned mesially; the maxilla with a low dorsal flange and a prominent anterior flange; the hyomandibula with enlarged anterior and posterior flanges and an extensive fossa and associated processes (absent in *Catostomus*); the dorsolateral ridge of the pterotic is nearly vertical; the preopercle is deep at the center, approaching half-moon shape; the lips are large and fleshy with large notches at the junction of the upper and lower lips, a shallow notch between the lower lips, a prominent cartilaginous ridge on the lower jaw, more numerous gill rakers; and smaller body size than *Catostomus* (*sensu stricto*). *Pantosteus columbianus* is intermediate between *Pantosteus* and *Catostomus* in many of the above characters; *P. plebeius* and *P. nebuliferus* are intermediate in the lip characters [20], [34]. Phylogenetic analysis of catostomins with 87 morphological and osteological traits (deposited in Dryad, doi:10.5061/dryad.51mm0) provided a well resolved tree (Fig. 4, modified from [20]), with bootstrap analysis providing strong support (>80%) for many deeper nodes, especially *Pantosteus*. The MP tree from the morphological analysis presented in [20] was deposited in TreeBASE, accession number TB2:S15337, (http://purl.org/phylo/treebase/phylows/study/TB2:S15337).

Phylogenetic analysis of mtDNA variation among taxonomic lineages as previously constituted are not consistent with monophyly of the genera, or monophyly of the *P. platyrhynchus* group, *P. discobolus*, or *P. columbianus*. The length of internodal branches (and concomitant bootstrap values) defining each of the mtDNA lineages that render these taxa as non-monophyletic are sufficiently large that sorting of mtDNA lineages is not a likely explanation for these incongruent results. Therefore, this conflict could be the result of convergence in morphological traits or introgressive transfer of mtDNA [35]. Both processes were hypothesized in previous comparative studies to explain similar patterns of morphological and biochemical characters [34], [41], [43]. Evaluation of these conflicts involves the use of cladistic analysis (Fig. 4) and fossils [20] to provide historical perspective, comparison with cases of present-day introgressive hybridization and statistical evaluation of phylogenetic trees. The 10 skull, jaw-bone, lip, and gill-raker traits are sufficiently numerous, anatomically diverse, and congruent, leading to high bootstrap values on the morphological tree (Fig. 4). Therefore, morphological homoplasy is considered unlikely. Because conflicting nodes are highly supported in both analyses, the most likely explanation for this discordance is introgression.

Regardless of the reasons for incongruence, some major revisions in the current taxonomy are required by the molecular data. Given the complicated nomenclatorial history of this group, we provide a summary of names we are resurrecting (Table 2) as well as morphological support for these designations. This is presented in the context of mtDNA and morphological phylogenies (Figs. 3, 4), which reveal examples of paraphyly involving *Catostomus* and *Pantosteus* as well as some of the lineages within *Pantosteus*.

**Table 2:** Proposed changes to the taxonomy of *Pantosteus* species.

original taxonomy	changed taxonomy	distribution
*Pantosteus clarkii*	unchanged	Lower Colorado River Basin
*P. columbianus*	unchanged	Columbia River Basin
*P. discobolus*	*P. discobolus*	Colorado River Basin
*P. discobolus*	*P. virescens*	northern Bonneville and upper Snake River basins
*P. discobolus jarrovii*	unchanged	upper Little Colorado River
*P. nebuliferus*	unchanged	Nazas and Aguanaval basins
*P. platyrhynchus*	*P. bondi*	Columbia River Basin (minus upper Snake)
*P. platyrhynchus*	*P. jordani*	Missouri River Basin
*P. platyrhynchus*	*P. lahontan*	Lahontan Basin
*P. platyrhynchus*	*P. platyrhynchus*	Bonneville, upper Snake and Green River basins
*P. plebeius*	unchanged	Grande, Guzman, Conchos, and Yaqui basins
*P. santaanae*	unchanged	Los Angeles region

### Interpretation and Diagnosis of Species

Morphological and molecular results are mostly consistent with previous literature for several species (*P. clarkii*, *P. santaanae*, *P. plebeius* and *P. nebuliferus*). Diagnoses and discussion for each of these species are presented below; see also [20], [34]. The remaining lineages, *P. columbianus* and those formerly referred to as *P. discobolus* and *P. platyrhynchus*, exhibit conflict between morphological and molecular traits that are considered in detail, along with hypotheses to explain the complicated polymorphisms and mixtures of traits among populations in the following sections.

*Pantosteus clarkii* (Baird and Girard) inhabits tributaries to the Lower Colorado Basin, including the Gila, Salt, Bill Williams, Pluvial White and Virgin rivers, which were connected to the Lower Colorado Basin below Grand Wash, but isolated from the Upper Colorado Basin and *P. discobolus* prior to the connecting flow through Grand Canyon at about 4.8 Ma [59], [60]. *Pantosteus clarkii* shares most of its morphological traits and molecular sequences with *P. santaanae*, *P. discobolus*, and *P. virescens*. MtDNA data presented here support the hypothesis that isolation of these populations is relatively recent. Examples of introgression between *P. clarkii* and *P. discobolus* and between *P. clarkii* and *P. platyrhynchus* have been found in samples from the Virgin River and Shoal Creek (Sevier drainage), respectively [34] (and Secor and Dowling, unpub. data).

*Pantosteus santaanae* Snyder is a generalized, small-bodied mountain sucker found on the Los Angeles Plain, geographically isolated from *P. clarkii* in the Lower Colorado Basin. Osteologically, it shares features with *P. clarkii* consistent with the ML tree; however, these are plesiomorphic, consistent with its position in the MP tree.

*Pantosteus plebeius* (Baird and Girard) is found in the Rio Grande Basin and associated internal drainages, as well as headwaters of adjacent Pacific coastal drainages in Mexico. It and its sister species, *P. nebuliferus*, comprise the sister group to the *discobolus*-*virescens*-*clarkii*-*santaanae* clade. Analysis of allozymes also identifies another distinct lineage in the Rio Mesquital, Mexico, that was not sampled here; additional sampling and characterization is therefore required [61].

*Pantosteus nebuliferus* (Garman) is found in the Nazas and Aguanaval basins, although mtDNA of this form is also found in the population in the Rio Miravalles of the Rio Piaxtla drainage. This suggests that *P. nebuliferus* is either more widespread than first thought, or populations in the Rio Piaxtla are introgressed.

*Pantosteus discobolus* (Cope) was described from the Green River in the Upper Colorado Basin. The divergent mtDNA of *P. virescens* of the Bonneville Basin and Upper Snake River forms, previously included in *P. discobolus*, is consistent with the depth of the caudal peduncle and other unique morphological features. These differences suggest recognition of the name *P. virescens* Cope. Therefore, we follow [40] in separating *P. virescens* (Weber, Bear and Upper Snake rivers) from *P. discobolus* of the Upper Colorado Basin (see below). *Pantosteus discobolus*, *P. virescens*, *P. clarkii*, *P. santaanae* and *P. plebeius* form a clade diagnosed by mtDNA as well as morphological traits [20]. As constituted here, *P. discobolus* is the large mountain sucker (adults 200–350 mm standard length) of the larger rivers in the Upper Colorado Basin. *Pantosteus virescens* (Cope in Cope and Yarrow) was based on a type specimen said to be from the San Juan drainage in Colorado, but [40] concluded that the type locality was mislabeled and *P. virescens* was identical to specimens that he collected from the Weber River, Utah, and the Bear River, Wyoming, both tributary to the northern Bonneville Basin. The distribution of *P. virescens* is consistent with that of other species in the Bonneville Basin and Upper Snake River: multiple connections existed between the Bonneville Basin and the Upper Snake River in the late Pleistocene with several fish species being exchanged at that time [7]. Similarly, stream captures between the upper Green River and the Upper Snake River have been postulated based on geological [62], [63] and fish evidence [2], yet the upper Green River populations have mtDNA of *P. discobolus*, despite possible influence from *P. virescens* and *P. platyrhynchus*. *Pantosteus virescens* was formerly abundant as the largest of the mountain suckers in the Weber, Bear, and Upper Snake rivers, but it is rare or absent in most former habitats, and modern specimens are smaller in size, especially in introgressed populations. Populations of *P. virescens* in the Upper Snake River drainage morphologically introgressed into *P. platyrhynchus* (p. 108 and Fig. 22 of [34]). Thus, the small extant *P. virescens* populations present in the Weber and Bear rivers should be provided high conservation significance as they represent the only remaining non-introgressed populations of this dwindling species.

The name *P. discobolus jarrovii* (Cope) has been assigned to a complex of ancient Arizona and New Mexico lineages that share mixed molecular, fin-ray, lip, and jaw characteristics of *P. discobolus* and *P. plebeius*. They are found in headwaters of the Little Colorado River in northeastern Arizona and northwestern New Mexico. The populations possess molecular, morphological and allozyme traits that originated in two separate but adjacent drainages, but which have been variously transferred in the Pleistocene via stream captures [34], [41], [64]. The populations are diverse and threatened with extinction. Their complex patterns of introgressed molecular, morphological and osteological characters are being described elsewhere to deal with controversial but critical conservation issues (Dowling et al., unpublished data).

*Pantosteus platyrhynchus* (Cope) was previously recognized as widely distributed throughout the Lahontan and Bonneville basins, Columbia and Snake rivers, and the headwaters of the Missouri drainage and Upper Colorado Basin; however, high mtDNA divergence among several lineages from these regions suggests that the taxonomy requires revision to more accurately reflect the apparent diversity. Former populations of *P. platyrhynchus* in the Missouri drainage are here referred to *P. jordani* Evermann; those from the Lahontan Basin are recognized as *P. lahontan* Rutter; and those from the Columbia-Lower Snake rivers were described as *P. bondi* [20].

*Pantosteus platyrhynchus*, as here diagnosed, occupies the Bonneville drainage and its former connective, the Upper Snake river, as well as small headwater streams in the Green River. The mtDNA of *P. platyrhynchus* in the Bonneville Basin is derived from *P. discobolus* (Fig. 3). This reflects the non-congruence of morphological traits, which are otherwise allied to northern populations in the Columbia River, Lahontan Basin, and Missouri drainage rather than *P. discobolus*. In addition, *P. platyrhynchus* contains two forms of mtDNA haplotypes diverged after acquisition of *P. discobolus* mtDNA with consistent slight morphological differences. One form occurs in the Sevier River (southern Bonneville Basin) and the other in the northern Bonneville Basin and Upper Snake River.

There is evidence for introgression between *P. platyrhynchus* and sympatric *Pantosteus* species. Populations in the Upper Snake and Green rivers are included in *P. platyrhynchus* based on the majority of their morphological traits; however, they have *P. virescens* and *P. discobolus* mtDNA, respectively, and mixtures of morphological traits of those species [34]. Broader sampling across this region is required to determine whether mtDNA introgression is partial or complete. The geographic pattern of variation in the number of predorsal scales suggests introgression between populations of *P. platyrhynchus* in Shoal Creek (Bonneville Basin) and *P. clarkii* introduced from the Virgin River (Secor and Dowling, unpublished data).

*Pantosteus jordani* Evermann is a small-bodied species from the upper Missouri drainage from the Black Hills of South Dakota to western Wyoming, Montana and Alberta, with Pleistocene fossils from western Kansas [20]. It is similar to *P. platyrhynchus*, with which it was formerly combined.

*Pantosteus lahontan* Rutter occupies the Lahontan Basin of Nevada, Oregon and California. It is similar to *P. platyrhynchus*, but has a slenderer hyomandibula, 30% as broad as long, with a cup-like post-dorsal process surrounding the post-dorsal fossa of the hyomandibula. It has 42–47 predorsal scales, usually 39–42 post-Weberian vertebrae, and 25–35 gill rakers on the outer row of the first arch [20].

*Pantosteus bondi* (Smith, Stewart, and Carpenter) occurs in the Columbia drainage, based upon samples from the Willamette, Similkameen, Boise and Salmon Falls drainages. This species has formerly been referred to as either *P. jordani* or *P. platyrhynchus* (see synonymy in [34]). It is diagnosed from *P. platyrhynchus* by its reciprocally monophyletic mtDNA and by its combination of a continuous but slightly emarginate ridge anterior to the opercular condyle of the hyomandibula, prominent post-dorsal fossae and process at the post-dorsal tip of the pterotic process of the hyomandibula, prominent post-mesial flange of the hyomandibula overlapping the antero-dorsal tip of the preopercle, medium width of the hyomandibula (about 34% as wide as long) and 29–37 gill rakers in the external row on the first gill arch [20].

*Pantosteus columbianus* Eigenmann and Eigenmann has a mixture of morphological traits of *P. virescens* and a species of *Catostomus*. Its mtDNA is most similar to that of *C. tahoensis* of the Lahontan Basin, suggesting hybrid origin involving that species. This interpretation requires immigration of *C. tahoensis* into the Snake River drainage, possibly at a time of Plio-Pleistocene spillovers from the Lahontan Basin to the Lower Snake River documented by [65], where it was apparently introgressed and absorbed into the populations of *P. virescens* that formerly occupied the Lower Snake River (according to fossil evidence, described below). Morphologically, *P. columbianus* is intermediate between those species in its lip shape and in many osteological traits, but shares scale counts and shapes of the jaws and suspensorium with *P. virescens* rather than *C. tahoensis* [20], [34].

### Dating mtDNA Lineages

Fossils provided bases for calibration points for two nodes in the tree, 12.7 Ma for the node at the base of *P. nebuliferus*, *P. plebeius*, *P. virescens*, *P. discobolus*, *P. clarkii*, and *P. santaanae*, and 5.5 Ma for the node ancestral to the clade of *P. virescens*, *P. discobolus*, *P. clarkii*, and *P. santaanae*. These are calibration points based on corrections of fossil ages [54]. The pertinent data on the fossils are as follows. The 12.7 calibration point is based on an oldest fossil age of 11.6 Ma–a synapomorphic postero-lateral ridge of a hyomandibula, which is shared with the clade including *P. discobolus, P. virescens, P. clarkii, P. santannae, and P. plebeius* (Figs. 3, 4). The key specimen (among three, 8.5–11.6 Ma) is the holotype hyomandibula of *P. hyomyzon* (UO F-55749) from UO locality 2337 in the Juntura Formation, Malheur County, Oregon, constrained by the overlying ash dated 11.5 Ma [20]. The number of horizons is seven.

The 5.5 Ma calibration point is based on a 4.5 Ma synapomorphic, broad and obtusely-angled shape of the fourth Weberian rib of *P. oromyzon*, this apomorphy is shared with the clade including *P. discobolus, P. virescens, P. clarkii, and P. santannae* (Figs. 3, 4). The key specimen is the holotype (one of two specimens of) *P. oromyzon* (UMMP 42356) from the basal Glenns Ferry Formation, Owyhee County, Idaho, constrained by the overlying Glenns Ferry oolite estimated to be 4.5 Ma [20]. The number of horizons is three. The fossil is approximately the same age as *P. asitus* of this clade, a dentary and other *Pantosteus* bones, from White Narrows, Clark County, Nevada [20]. We do not include fossil *P. columbianus* in the calibration of molecular rates because the hybrid origin of this taxon causes discordance between morphological and mtDNA topologies. Other populations have not been introgressed to this extent and estimated divergence times are offered as hypotheses with some support in the form of geological and hydrologic correlations.

**Figure 5:**
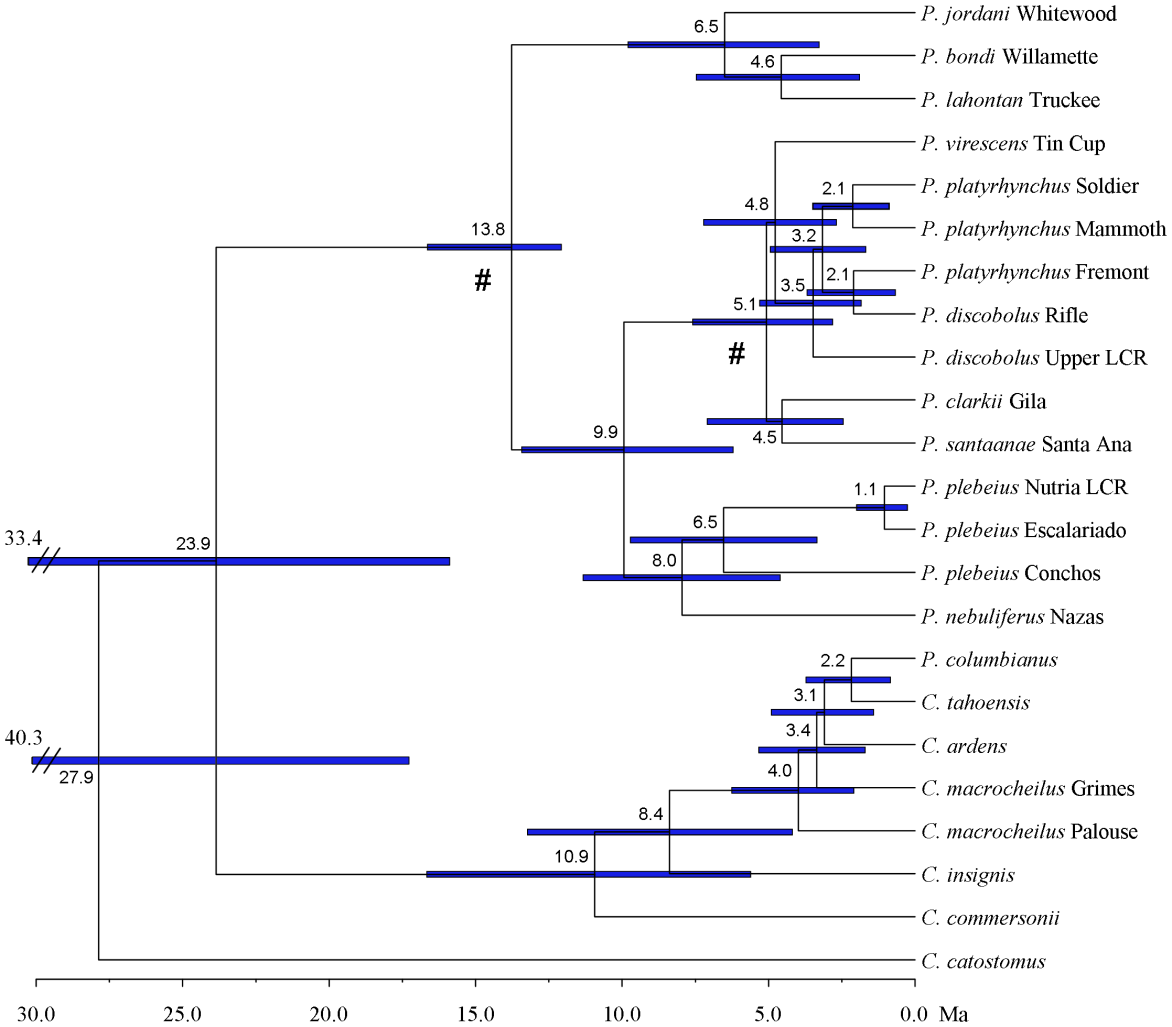
Bayesian molecular clock estimates for *Pantosteus* based on analysis of mitochondrial DNA. Horizontal bars represent the 95% highest posterior density ranges. Locality details are provided in Table 1, OTU order and labels match those in Fig. 3.

We explored a variety of subsets of our sequence dataset to examine the effects of sample selection on divergence estimates, primarily to determine the effect of including outgroup samples from *Catostomus* on our estimates within *Pantosteus*. In all cases the results were similar (within 10%, results not shown). Sequences from sixteen individuals were selected to represent the phylogenetic diversity found within *Pantosteus* along with seven appropriate outgroup samples within *Catostomus*. The BEAST analysis was constrained to fit the ML topology (Fig. 3), with dates based on their mean and 95% highest posterior density (HPD) presented in Fig. 5; all parameter estimates had effective sample sizes greater than 2600. Each of the major nodes and associated geological events are discussed in detail below. These estimates of divergence time are based on mtDNA; therefore, they may be influenced by introgression and must be interpreted with this caveat in mind. In cases where extensive introgression of morphology and mtDNA have occurred (e.g., *P. discobolus jarrovii*, *C. columbianus*), this method provides an estimate of the date at which this mtDNA lineage was transferred into that form, allowing for inferences of the age of hybridization events.

The estimated mean time of divergence between *Pantosteus* and *Catostomus* (exclusive of *C. catostomus*) was 25.1 Ma (95% HPD of 35.3–16.6 Ma) [4]. This corresponds to a period of high mountains throughout the area that was in the early process of becoming the Basin and Range Province. The location of the earliest fossils in the proto-Columbia Basin, which corresponds with both high fossil and extant catostomid diversity in the West, suggests that the northern Great Basin is a plausible region in which initial divergence took place. The middle Miocene Nevada-Washington rift and the development of the Oregon-Idaho Graben in southern Oregon [66] represent major tectonic structures and events possibly related to this time and place of early catostomin evolution.

**Figure 6:**
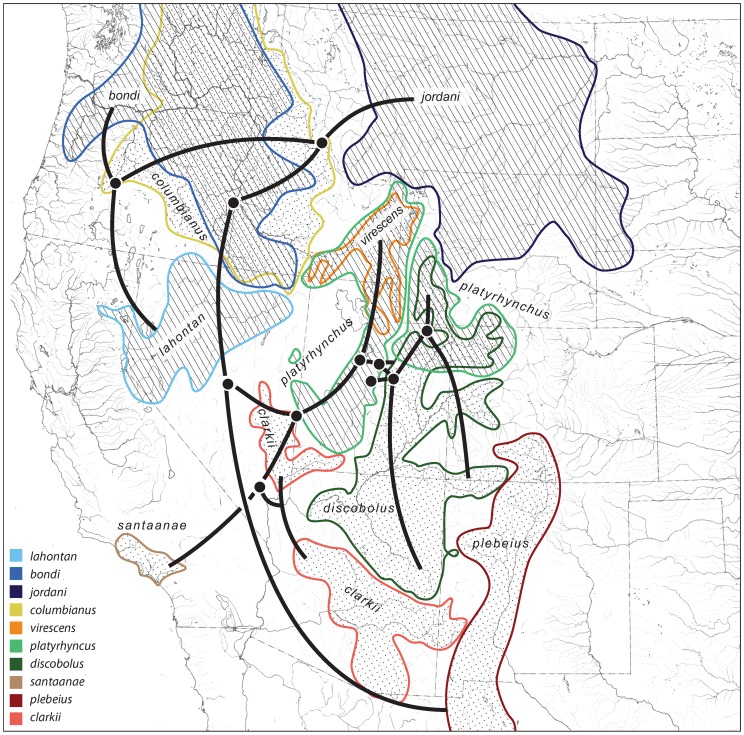
*Pantosteus* species distribution patterns with overlay of the species relationships from Fig. 5. Estimated ages of branching events are in Fig. 5.

The three oldest fossils of *Pantosteus*, 11.6, 10.3 and 8.5 Ma, are from separate drainages in central Washington and southeast Oregon [20], at the northern edge of the Basin and Range Province [3]. The estimated age of first occurrence of the clade bearing synapomorphies of these fossils (Fig. 5, mean 14.5 Ma, 95% HPD of 17.3–12.8 Ma) coincides with the extensional opening up of the Basin and Range topography. Because nearly all of the taxa in *Pantosteus* are in or adjacent to either the northern or southern ends of the Great Basin (Fig. 2, 6), it is probable that the distribution and differentiation of these fishes owes its history to habitats and barriers created in this geological province. *Pantosteus* inhabit medium-gradient streams of the size that flow from the hundreds of north-south trending mountain ranges of the Basin and Range province (Fig. 1). These mountains also form barriers that isolate populations and initiate differentiation, but this differentiation is periodically interrupted by moist-dry climatic cycles that allow dispersal through renewed aquatic connections [26].

The relationship between geography and phylogenetic relationships based on mtDNA is presented in Fig. 6. The oldest branches of *Pantosteus*, based on molecular analyses, are (1) the broad *P. plebeius*-*P. discobolus* clade, distributed south through the Great Basin to Mexico, including the upper Rio Grande Basin in the Rio Grande rift, and onto the Colorado Plateau, and (2) the *P. platyrhynchus* group, distributed in the north from the Columbia drainage and northern Great Basin to the upper Missouri drainage. Our analyses suggest that the first great pathway of dispersal and divergent evolution, following the initial split, was south through the Great Basin.

In the Basin and Range province, the *P. plebeius* and *P. discobolus* clades began diverging around 10.5 Ma (Fig. 5, 95% HPD of 13.9–6.6 Ma), a time of tectonic, volcanic, and climatic changes in the northern Great Basin [3], [4], [66]. The *P. discobolus* clade differentiated into its five modern species in the northern Great Basin and Colorado Plateau about 5.4 Ma (Fig. 5, 95% HPD of 8.1–3.1 Ma). The Upper and Lower Colorado basins achieved their connection through Grand Canyon at 4.8 Ma [59], [60], so it is likely that differentiation was occurring in pre-existing drainages in the Grand Wash area [19], [67], [68] between 10 and 5 Ma. At this time, the Colorado Plateau was elevating but probably had less *Pantosteus* habitat than the Great Basin [2], [11], [69]. Four of the five species in the *P. discobolus* group dispersed through and differentiated in the Great Basin west of the emerging Colorado Plateau (Figs. 1, 6).

The Bonneville and Lahontan basins, in the eastern and western halves of the Great Basin, were extending in the late Miocene, causing numerous north-south drainage connections. The Colorado River was in a stage preceding the modern Grand Canyon at this time, although older canyons were present [68],[70],[71]. *Pantosteus* (and *Catostomus*) must have expanded their range through the Basin and Range area where the Colorado Plateau and the northern and southern portions of the Great Basin meet (Figs. 1, 6), to establish the southern clades in Mexico (see below) during this period.

The problem of the relationships of *P. santaanae* to either *P. clarkii* or to the remainder of this clade is not resolved by the phylogenetic analysis of mtDNA as indicated by conflict between ML and MP analyses. Morphological data are also ambiguous. An earlier date of divergence is plausible if the ancestors date back to the dispersal southward to Mexico. These estimates might be impacted, however, by their higher rates of sequence evolution (as evidenced by their long branch, Fig. 3) induced by warm temperatures and small body size [72].

The map and trees (Figs. 2, 3, 4, 5, 6) suggest that the *P. platyrhynchus* group originated 14.5 Ma (Fig. 5, 95% HPD of 17.3–12.8 Ma) and diversified over the last 6.9–4.8 Ma (Fig. 5, 95% HPD of 10.5–2.0 Ma) into four species: *P. bondi* in the Columbia drainage, *P. jordani* in the upper Missouri drainage, *P. lahontan* in the Lahontan Basin, and *P. platyrhynchus* in the Bonneville Basin, Upper Snake River and the Green River. This diversification corresponds to a time of major volcanic and tectonic activity in the Columbia region [73]. These lineages probably began in three pre-Columbia River drainages of the northern Basin and Range Province, where the three oldest *Pantosteus* fossils were recovered. All except one differentiated in the Northern Great Basin; that one exception, *P. jordani*, evolved initially in the Yellowstone region, now in the headwaters of the Missouri River, which at that time drained to Hudson Bay [74]. Later, in the Pleistocene, there were exchanges among the Bear, Snake and Green rivers east of the Snake River Plain (Figs. 1, 6) in southwest Wyoming [2], [6], [62], which were possibly responsible for the transfer of individuals of *P. platyrhynchus* from west to east (see below).

The northern Bonneville/Upper Snake and southern Bonneville populations of *P. platyrhynchus* last shared a common ancestor 2.3 Ma (Fig. 5, 95% HPD of 3.7–1.0 Ma). This separation between the southern and northern Bonneville Basin is a common pattern shared by two other fishes: *Lepidomeda copei* [75] and *Rhinichthys osculus* [76]. The Sevier Basin was isolated from the Salt Lake Basin by a barrier higher than the present, from at least 3 Ma until the early Pleistocene [77]. The recent connection of the southern and northern halves of the Bonneville Basin via high stands of Lake Bonneville appears to have not facilitated movement between them, which may be due to these species being stream specialists that are not typically found in lakes.

The divergence of Mexican and Rio Grande Basin *Pantosteus*, between 8.4 and 1.1 Ma (Fig. 5, 95% HPD of 11.8–0.3 Ma), suggest dispersal from Idaho southward through Nevada and Utah prior to this time (see [70]). The drainage changes responsible for divergence of *P. plebeius* and *P. nebuliferus* in central Mexico are not known.

### Importance of Introgressive Hybridization in *Pantosteus*

Comparison of mtDNA distributions among taxa with morphological data, biogeography and paleontology reveals noncongruence that suggests either extensive morphological convergence or introgressive transfer of mtDNA among lineages in the ancient past (Fig. 4). Several considerations suggest resolution in favor of the introgressive genetic transfer hypothesis. (1) Paleontological, morphological and biogeographical interpretations are often consistent with each other, but not with the mtDNA phylogeny. (2) The discrepancies in the mtDNA phylogeny involve taxa known to have experienced introgressive hybridization in recent times [34], thus allowing for the possibility of introgression occurring in the past. (3) Some examples of introgression among fishes of western North America have been observed in the fossil record [35] or have been verified by identification of geological evidence of stream capture consistent with the transfer leading to the introgression [41]. (4) Lastly, the broad scatter of morphological traits within individuals of an introgressed population show individually unique patterns of variation, suggesting different degrees of penetration of different alleles, epigenetic interactions and selective regimes. That is, there are unusually variable and unique patterns of assorted polymorphisms among individuals [34], [35], [78]–[80]. The principal examples of introgressive hybridization in *Pantosteus* are the Zuni Sucker (*P. discobolus jarrovii*), the Bonneville Basin, Snake River and Green River *P. platyrhynchus*, and *P. columbianus*, all of which demonstrate geographically patchy and non-concordant distributions of characters and alleles typical of introgression [37], [81], [82]. We now discuss each example of introgression below.

*Pantosteus plebeius* introgression with *P. discobolus* was documented with morphological, biogeographic and geologic data by [34], and the hypothesis tested and supported with biochemical data [41], [64]. The taxon, *P. discobolus jarrovii* Cope was based on the downstream cline of mixed and intermediate characters of the introgressed population, inferred to have resulted from a stream capture and fish transfer in the Pleistocene, with an estimated mean age of 1.1 Ma (Fig. 5, 95% HPD of 2.1–0.3 Ma). Because of multiple haplotypes, morphologies and conservation problems, these unusually complex populations are under separate investigation with more detailed sampling (Dowling at al., unpublished).

*Pantosteus discobolus* mtDNA has replaced that of *P. platyrhynchus* throughout its range as exhibited by the discordance of morphological and mtDNA characters. Morphological synapomorphies detailed by [34] and discussed above support the placement of *P. platyrhynchus* in the clade of populations, all formerly classified as *P. platyrhynchus*, distributed in the Columbia-Snake drainage, Missouri drainage, Green River and northern Great Basin. These populations are well known to hybridize with sympatric catostomins elsewhere [34], [79], so it is no surprise to find mtDNA of the *P. discobolus* group in *P. platyrhynchus* in the Upper Snake River and Green River. It was unexpected, however, to find all sampled individuals of Bonneville Basin and Upper Snake River *P. platyrhynchus* bearing *P. discobolus* mtDNA, dating from 3.4 Ma (Fig. 5, 95% HPD of 5.2–1.8 Ma). Introgression between these species in the Green River, and between *P. platyrhynchus* and *P. virescens* in the Upper Snake River, were described by [34]. However, no morphological traits of *P. discobolus* were observed in *P. platyrhynchus* populations in the Bonneville Basin. The presence of *P. discobolus* mtDNA in Bonneville Basin and Upper Snake River *P. platyrhynchus* populations is especially puzzling because *P. discobolus* is allopatric to Bonneville Basin *P. platyrhynchus*; *P. virescens* is the sympatric congener (Fig. 3). *Pantosteus discobolus* and *P. virescens* are larger fish, preferring larger, downstream reaches; *P. platyrhynchus* is a headwater species, but where stream habitats overlap they are sympatric. Additional sampling is required across the range of *P. platyrhynchus* to determine if patterns of introgression are ubiquitous.

The hypothesis that these populations of *P. platyrhynchus* are actually genetic *P. discobolus* that have evolved gill-raker, scale, fin, pigment, and osteological traits that converge on *P. platyrhynchus*, is rejected based on the diversity, congruence, and independence of the morphological evidence. This assumption of independence of diverse morphological traits should be tested, however, with studies of nuclear genes responsible for morphology [83].

Geomorphic evidence of stream capture, possibly responsible for the transfer of fish between the Snake and Green rivers in southwest Wyoming, is noted above. Preliminary sampling indicates that the introgressed traits, which seemed more or less stabilized when observed in the Snake and Green rivers by ([34], see graph, p. 109), had become more prevalent in samples examined for the present study. *Pantosteus platyrhynchus* samples in the Upper Snake River exhibit *P. virescens* morphological traits and mtDNA (Fig. 3) to varying degrees. *Pantosteus discobolus* mtDNA (Fig. 3) and morphological traits were scattered in *P. platyrhynchus* specimens in the Green River.

In particular, the *P. platyrhynchus* in Fremont Lake, in the upper Green River of Wyoming, has mtDNA that differs by a p-distance of 1.1% from *P. discobolus* collected in the upper Grand River at Rifle, Colorado (Fig. 3). The *P. discobolus* mtDNA introgressed into *P. platyrhynchus* is estimating the differentiation of *P. discobolus* in the Green River from populations in the upper Grand River (2.3 Ma, 95% HPD of 3.9–0.7 Ma, Fig. 5), not *P. platyrhynchus*.

*Pantosteus columbianus* appears to have a complex history of introgression between *C. tahoensis* and *P. virescens* ([20] and this paper) and a later history of introgression with *C. macrocheilus* [79]. *Pantosteus columbianus* was described originally in *Pantosteus* and later in *Catostomus* because of its mixture of diagnostic traits [20]. Its lips are intermediate, most of its osteological traits are diagnostic of *Pantosteus*, and its mtDNA is most similar to that of *C. tahoensis*. The genetic divergence between the mtDNA of *P. columbianus* and *C. tahoensis* implies a mean age of introgression of 2.3 Ma (Fig. 5, 95% HPD of 4.0–0.9 Ma). Fossils of *P. columbianus* in the western Snake River plain between 3 and 2 Ma help constrain the time of introgression that formed *P. columbianus*. The absence of *P. virescens* below the falls of the Snake River, notwithstanding fossil evidence of its occurrence there at 4.5 Ma, implies that the immigration of *C. tahoensis* during spillover from the Lahontan Basin to the Snake River drainage in the Pleistocene [65], resulted in complete genetic absorption of the two parental populations into a species of introgressed origin, *P. columbianus*. Further tests of this hypothesis with nuclear genes should be instructive. Sub-populations of *P. columbianus* are isolated in the headwaters of the Palouse River, Washington, the Wood River, Idaho, and the Deschutes and Malheur rivers, Oregon. Their morphological differences from the central populations suggest an early widespread form of the species that was more *Pantosteus*-like, especially in bones and lip structure. These indicate significant morphological change during their existence [34].

## Conclusions

The Basin and Range Province is a unique setting in which to study the effects of insular isolation on diversification of plants and of aquatic and terrestrial animals [65], [84], [85]. The fragmentation of a late Mesozoic mountain belt along the southwest margin of North America by crustal thinning and extension created hundreds of narrow valleys separated by hundreds of elongate mountainous remnants (Fig. 1). The valleys were frequently isolated except when filled and externally drained [65] and the mountains were isolated except when cool climates forced transitional and boreal life, including mountain suckers, down and out across valley floors. During late Neogene glacial/interglacial stages, fluctuations in barriers and connections led to about 20 cycles of isolation, differentiation, and repeated secondary genetic contact among populations of organisms. Only the most recent of these glacial and post-glacial stages is well studied.

The distribution of mtDNA among populations of *Pantosteus* reflects this cyclic history. Many instances of secondary contact resulted in hybridization, and some populations experienced introgression, or even amalgamation (Dowling et al., 1997 [86]), resulting in conflicting patterns in morphological and DNA interpretations of relationships. Most species of western catostomids [79] and cyprinids [78] show evidence of hybridization and all 11 species of *Pantosteus* have hybridized with sympatric relatives [34], [79]. Morphological and molecular evidence presented here suggests that introgression was ancient (e.g., pre-human influence) in *P. tahoensis*×*P. virescens* ( = *P. columbianus*), *P. platyrhynchus*×*P. discobolus*, *P. platyrhynchus*×*P. virescens*, *P. discobolus*×*P. clarkii*, *P. discobolus*×*P. plebeius* ( = *P. d. jarrovii*) and possibly *P. plebeius*×*P. nebuliferus*. Many other hybrid individuals, e.g., *P. platyrhynchus*×*P. clarkii* and many hybrids involving *Catostomus* [79], show distributions and characters that suggest recent hybridization in the presence of environmental disturbance and inter-basin transfer by humans. Environmental disturbance in the form of compromised spawning sites were presumably responsible for many ancient introgressions.

Ancient introgression causes the production of morphotype/haplotype combinations that yield misleading phylogenetic reconstructions [35] and misidentification of specimens by scientists and managers. Changes in biodiversity, negative or positive, are of considerable interest [86]–[88]. Loss of species is a possibility, as in the case of dwindling populations of *P. virescens* in the Upper Snake, Bear, and Weber rivers. Replacement of species, such as *C. tahoensis* and *P. virescens* by *P. columbianus* in the Columbia drainage is an interesting example. Post-glacial expansion of *P. columbianus* north to 55 degrees in Canada is evidence of an adaptable genome. Whether the added genetic variability in introgressed *P. platyrhynchus* in the Green River is adaptive, neutral, or harmful in its changing environment is not known, but the introgressed alleles have increased between 1963 [34] and the present study.

Demonstration of introgression has taxonomic as well as evolutionary implications. Smith et al. [20] noted that introgressive homoplasy renders *Pantosteus* and other catostomine genera polyphyletic and subsumed them in the genus *Catostomus* to maintain monophyly in the group. In the present paper, well-established names of genera are conserved in the interest of stability in the classification system and uniqueness of the clades. Comparative studies of population genetics, genomics and impact of diverse sources of genetic variation in populations of *P. discobolus jarrovii*, *P. platyrhynchus*, *P. lahontan*, *P. jordani*, and other catostomins will contribute to an understanding of the remarkable failure of selection to reduce heterospecific matings and introgression in this group.

## Supporting Information

Figure S1 All primers used to generate catostomid sequences.
(PPT)

Table S1 Mean between species p-distances as percentages (lower left) for each *Pantosteus* lineage, *Catostomus* as a whole, and the remaining outgroups as a whole.
(DOC)
