# Supplementary material for: Influence of Introgression and Geological Processes on Phylogenetic Relationships of Western North American Mountain Suckers (Pantosteus, Catostomidae)
Source: PLoS One. 2014 Mar 11;9(3):e90061. doi: 10.1371/journal.pone.0090061 (PMC3949674; doi:10.1371/journal.pone.0090061)
Supplement: Figure S1 — All primers used to generate catostomid sequences. (PPT) [file pone.0090061.s001.ppt]

## Slide 1
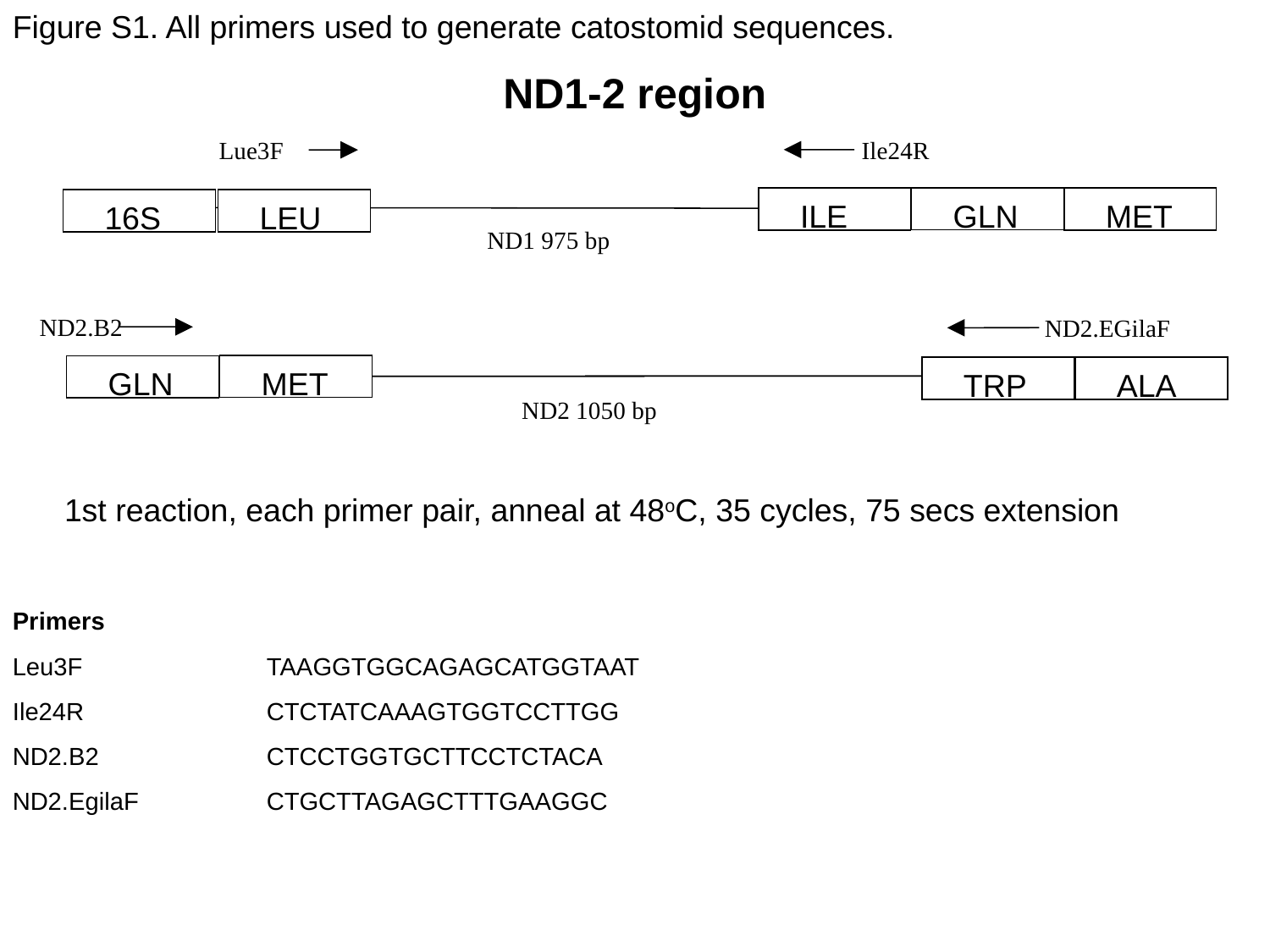

Figure S1. All primers used to generate catostomid sequences.
ND1-2 region
Ile24R
Lue3F
ILE
SER
GLN
MET
16S
LEU
ND1 975 bp
ND2.B2
ND2.EGilaF
MET
LEU
GLN
TRP
GLU
ALA
ALA
ALA
ALA
ND2 1050 bp
1st reaction, each primer pair, anneal at 48oC, 35 cycles, 75 secs extension
Primers
Leu3F		TAAGGTGGCAGAGCATGGTAAT
Ile24R		CTCTATCAAAGTGGTCCTTGG
ND2.B2		CTCCTGGTGCTTCCTCTACA
ND2.EgilaF		CTGCTTAGAGCTTTGAAGGC

## Slide 2
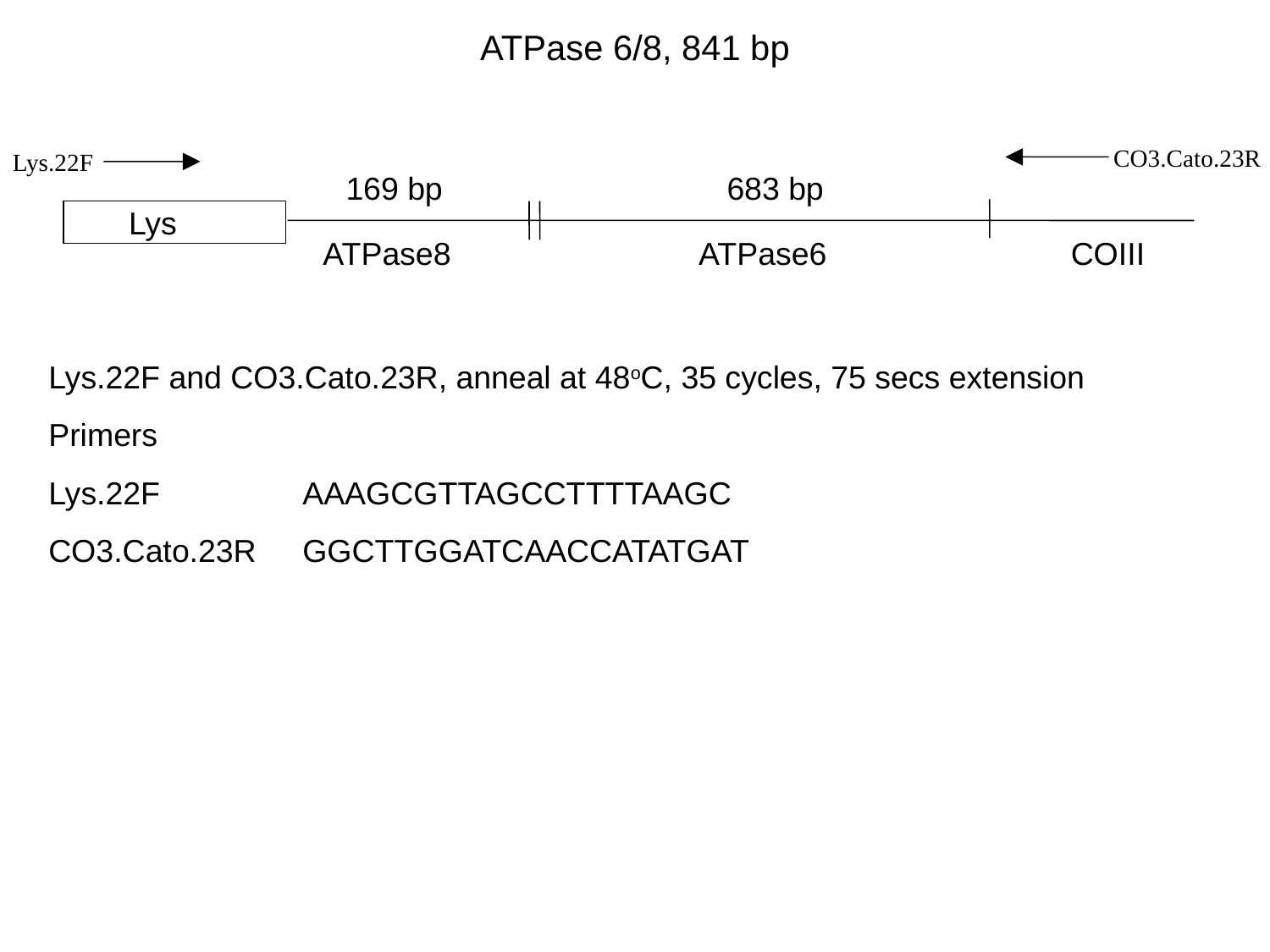

ATPase 6/8, 841 bp
CO3.Cato.23R
Lys.22F
169 bp			683 bp
Lys
ATPase8
ATPase6
COIII
Lys.22F and CO3.Cato.23R, anneal at 48oC, 35 cycles, 75 secs extension
Primers
Lys.22F		AAAGCGTTAGCCTTTTAAGC
CO3.Cato.23R	GGCTTGGATCAACCATATGAT

## Slide 3
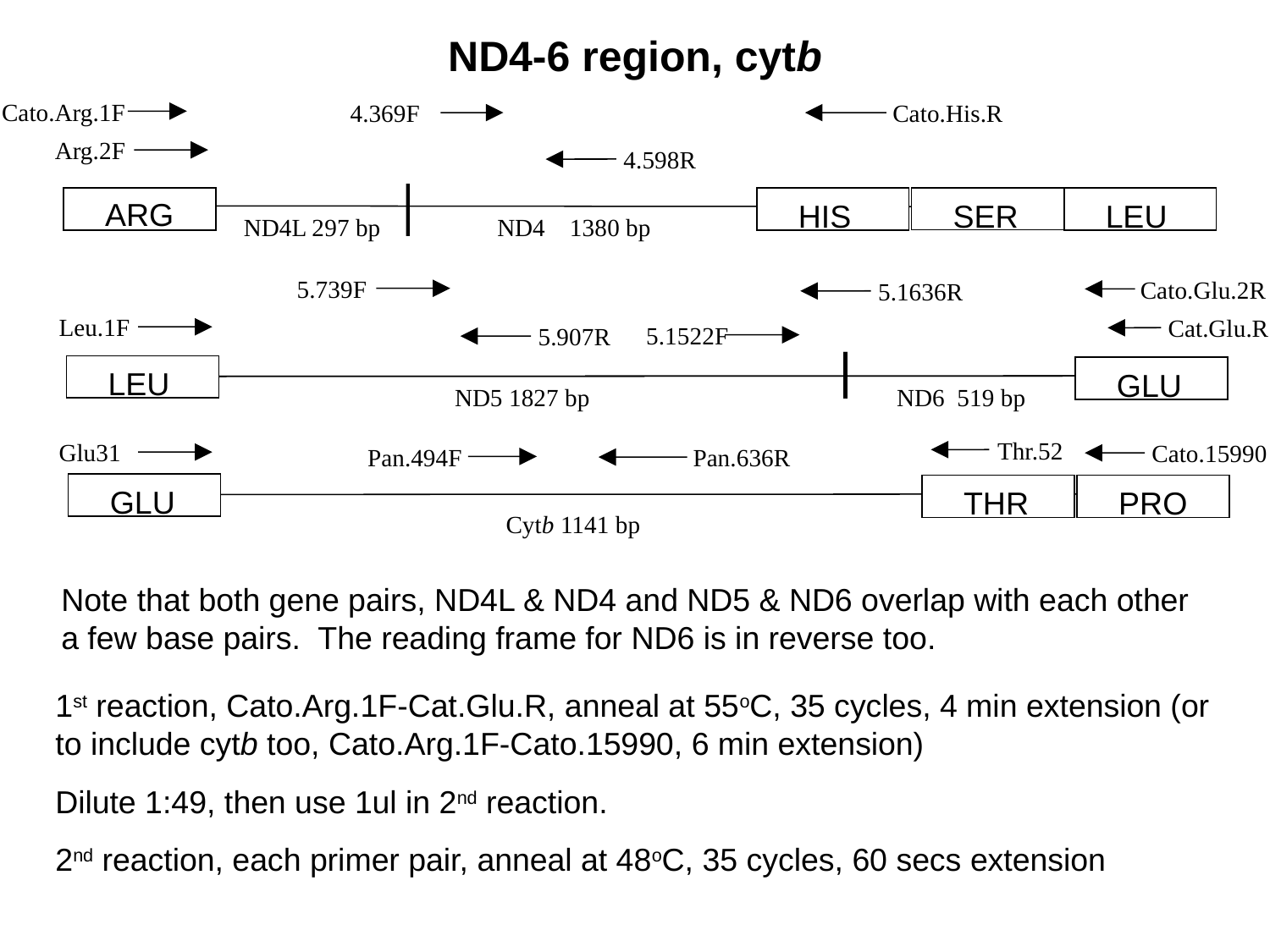

ND4-6 region, cytb
Cato.Arg.1F
4.369F
Cato.His.R
Arg.2F
4.598R
ARG
HIS
SER
SER
LEU
ND4L 297 bp ND4 1380 bp
5.739F
Cato.Glu.2R
5.1636R
Leu.1F
Cat.Glu.R
5.1522F
5.907R
LEU
GLU
LEU
GLU
ND5 1827 bp ND6 519 bp
Thr.52
Glu31
Cato.15990
Pan.494F
Pan.636R
GLU
THR
PRO
Cytb 1141 bp
Note that both gene pairs, ND4L & ND4 and ND5 & ND6 overlap with each other a few base pairs. The reading frame for ND6 is in reverse too.
1st reaction, Cato.Arg.1F-Cat.Glu.R, anneal at 55oC, 35 cycles, 4 min extension (or to include cytb too, Cato.Arg.1F-Cato.15990, 6 min extension)
Dilute 1:49, then use 1ul in 2nd reaction.
2nd reaction, each primer pair, anneal at 48oC, 35 cycles, 60 secs extension

## Slide 4
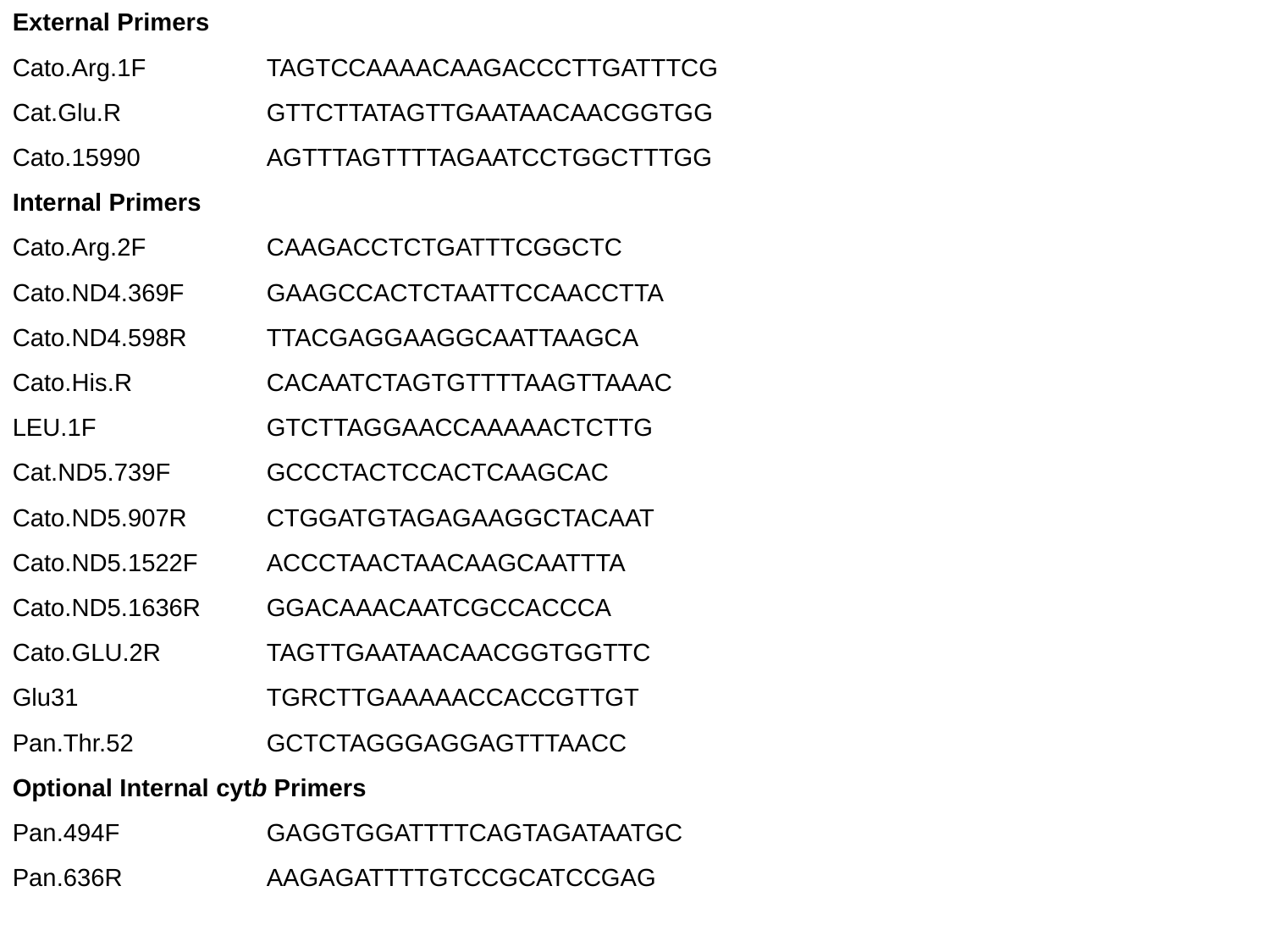

External Primers
Cato.Arg.1F	TAGTCCAAAACAAGACCCTTGATTTCG
Cat.Glu.R		GTTCTTATAGTTGAATAACAACGGTGG
Cato.15990	AGTTTAGTTTTAGAATCCTGGCTTTGG
Internal Primers
Cato.Arg.2F	CAAGACCTCTGATTTCGGCTC
Cato.ND4.369F	GAAGCCACTCTAATTCCAACCTTA
Cato.ND4.598R	TTACGAGGAAGGCAATTAAGCA
Cato.His.R		CACAATCTAGTGTTTTAAGTTAAAC
LEU.1F  		GTCTTAGGAACCAAAAACTCTTG
Cat.ND5.739F	GCCCTACTCCACTCAAGCAC
Cato.ND5.907R	CTGGATGTAGAGAAGGCTACAAT
Cato.ND5.1522F	ACCCTAACTAACAAGCAATTTA
Cato.ND5.1636R	GGACAAACAATCGCCACCCA
Cato.GLU.2R	TAGTTGAATAACAACGGTGGTTC
Glu31		TGRCTTGAAAAACCACCGTTGT
Pan.Thr.52		GCTCTAGGGAGGAGTTTAACC
Optional Internal cytb Primers
Pan.494F		GAGGTGGATTTTCAGTAGATAATGC
Pan.636R		AAGAGATTTTGTCCGCATCCGAG
