# Supplementary material for: Influence of Introgression and Geological Processes on Phylogenetic Relationships of Western North American Mountain Suckers (Pantosteus, Catostomidae)
Source: PLoS One. 2014 Mar 11;9(3):e90061. doi: 10.1371/journal.pone.0090061 (PMC3949674; doi:10.1371/journal.pone.0090061)
Supplement: Table S1 — Mean between species p-distances as percentages (lower left) for each Pantosteus lineage, Catostomus as a whole, and the remaining outgroups as a whole. (DOC) [file pone.0090061.s002.doc]

Supplementary Table 1. Mean between species p-distances as percentages (lower left) for each *Pantosteus* lineage, *Catostomus* as a whole, and the remaining outgroups as a whole.

|  |  | 1 | 2 | 3 | 4 | 5 | 6 | 7 | 8 | 9 | 10 | 11 | 12 | 13 | 14 |
| --- | --- | --- | --- | --- | --- | --- | --- | --- | --- | --- | --- | --- | --- | --- | --- |
| *P. jordani* | 1 |  |  |  |  |  |  |  |  |  |  |  |  |  |  |
| *P. bondi* | 2 | 4.2 |  |  |  |  |  |  |  |  |  |  |  |  |  |
| *P. lahontan* | 3 | 5.4 | 5.0 |  |  |  |  |  |  |  |  |  |  |  |  |
| *P. virescens* | 4 | 7.2 | 7.2 | 8.6 |  |  |  |  |  |  |  |  |  |  |  |
| *P. platyrhynchus*, Sth Bonneville | 5 | 7.8 | 7.7 | 9.0 | 3.5 |  |  |  |  |  |  |  |  |  |  |
| *P. platyrhynchus*, Nth Bonneville | 6 | 7.9 | 8.1 | 9.1 | 4.0 | 3.1 |  |  |  |  |  |  |  |  |  |
| *P. discobolus*, Colorado | 7 | 7.3 | 7.2 | 8.7 | 2.4 | 2.7 | 3.0 |  |  |  |  |  |  |  |  |
| *P. discobolus*, Little Colorado R | 8 | 7.6 | 7.4 | 8.8 | 3.1 | 3.5 | 3.6 | 2.2 |  |  |  |  |  |  |  |
| *P. clarkii* | 9 | 7.2 | 7.1 | 8.6 | 2.7 | 3.5 | 3.9 | 2.5 | 3.2 |  |  |  |  |  |  |
| *P. santaanae* | 10 | 8.9 | 9.1 | 10.1 | 5.5 | 6.1 | 6.4 | 5.2 | 5.8 | 5.1 |  |  |  |  |  |
| *P. plebeius*, Rio Grande/Guzman | 11 | 8.7 | 8.9 | 9.7 | 6.9 | 7.4 | 7.6 | 6.9 | 7.1 | 7.0 | 8.4 |  |  |  |  |
| *P. nebuliferus* | 12 | 9.7 | 9.7 | 10.3 | 8.0 | 8.6 | 8.8 | 8.1 | 8.5 | 8.2 | 9.4 | 8.7 |  |  |  |
| *P. plebeius*, Pacific Mexico | 13 | 8.3 | 8.2 | 9.5 | 5.6 | 6.4 | 6.6 | 5.4 | 5.9 | 5.7 | 7.5 | 6.5 | 8.3 |  |  |
| *Catostomus* | 14 | 12.4 | 12.6 | 13.4 | 11.7 | 12.2 | 12.3 | 11.6 | 11.9 | 11.4 | 12.3 | 12.8 | 13.4 | 12.3 |  |
| outgroups | 15 | 18.3 | 18.4 | 19.0 | 17.7 | 18.2 | 18.3 | 17.6 | 17.7 | 17.5 | 18.1 | 18.5 | 19.2 | 17.9 | 16.6 |
